# Quantitative Proteomics to Characterize Specific Histone H2A Proteolysis in Chronic Lymphocytic Leukemia and the Myeloid THP-1 Cell Line

**DOI:** 10.3390/ijms15069407

**Published:** 2014-05-27

**Authors:** Pieter Glibert, Liesbeth Vossaert, Katleen Van Steendam, Stijn Lambrecht, Filip Van Nieuwerburgh, Fritz Offner, Thomas Kipps, Maarten Dhaenens, Dieter Deforce

**Affiliations:** 1 Laboratory of Pharmaceutical Biotechnology, Ghent University, 72 Harelbekestraat, B-9000 Ghent, Belgium; E-Mails: pieter.glibert@ugent.be (P.G.); liesbeth.vossaert@ugent.be (L.V.); katleen.vansteendam@ugent.be (K.V.S.); filip.vannieuwerburgh@ugent.be (F.V.N.); maarten.dhaenens@ugent.be (M.D.); 2 Department of Rheumatology, Ghent University Hospital, 185 1P7 De Pintelaan, B-9000 Ghent, Belgium; E-Mail: stijn.lambrecht@hotmail.com; 3 Department of Hematology, Ghent University Hospital, 185 1P7 De Pintelaan, B-9000 Ghent, Belgium; E-Mail: fritz.offner@ugent.be; 4 Department of Medicine, Moores Cancer Center, University of California at San Diego (UCSD), 3855 Health Sciences Drive, La Jolla, CA 92093, USA; E-Mail: tkipps@ucsd.edu

**Keywords:** histone H2A, proteolysis, histone clipping, chronic lymphocytic leukemia, THP-1 cells, quantitative proteomics

## Abstract

Proteome studies on hematological malignancies contribute to the understanding of the disease mechanism and to the identification of new biomarker candidates. With the isobaric tag for relative and absolute quantitation (iTRAQ) method we analyzed the protein expression between B-cells of healthy people and chronic lymphocytic leukemia (CLL) B-cells. CLL is the most common lymphoid cancer of the blood and is characterized by a variable clinical course. By comparing samples of patients with an aggressive *vs.* indolent disease, we identified a limited list of differentially regulated proteins. The enhanced sensitivity attributed to the iTRAQ labels led to the discovery of a previously reported but still not clarified proteolytic product of histone H2A (cH2A) which we further investigated in light of the suggested functional properties of this modification. In the exploratory proteome study the Histone H2A peptide was up-regulated in CLL samples but a more specific and sensitive screening of a larger patient cohort indicated that cH2A is of myeloid origin. Our subsequent quantitative analysis led to a more profound characterization of the clipping in acute monocytic leukemia THP-1 cells subjected to induced differentiation.

## 1. Introduction

Proteomics approaches are often trailing genetic studies but are essential in the multi-disciplinary field of hematological research. As opposed to, e.g., mRNA microarray data, there is a better understanding of which proteins are actually expressed, although seeing the forest for the trees in long lists of protein identifications remains challenging [1,2]. In neoplastic hematology, protein studies have contributed to the elucidation of the disease mechanism, defined prognostic or therapeutic biomarkers and clarified previously reported uncharacterized phenomena [2]. By analyzing body fluids, cell lines, and tissues with quantitative high throughput mass spectrometry techniques complementary biological insights in hematopoietic malignancies can be generated. Uncovering relevant posttranslational modifications (PTMs), such as phosphorylations and proteolytical cleavages, might be associated with specific disease stages and could, hence, be informative on the biology of the disease [3].

The most common adult hematopoietic malignancy is chronic lymphocytic leukemia (CLL), a disease characterized by a widely variable median survival. After the initial staging of the patient, the risk of progression is defined by a set of genetic and protein based laboratory assays. A well-established prognostic marker is the mutational status of the immunoglobulin heavy chain (*IGVH*) genes encoding for the B-cell antigen-binding domain: CLL patients who have B-cells with unmutated (UM, >98% germ line identity) *IGVH* genes have an unfavorable outcome, whereas mutated (M) *IGHV* genes predict a more indolent course [4]. Surrogate markers on the protein level, such as the Zeta-chain-associated protein kinase (ZAP) 70 and CD38, are more easily applicable in the clinical practice although CD38 is considered to have less predictive value since discordancy with gene status is commonly observed and problems with standardization occur. ZAP70 expression however, is still used in the research on CLL pathogenesis and is considered as an independent biomarker [5]. Hence, for determining disease progression and survival of CLL patients, specific genetic markers, e.g., chromosomal aberrations, such as 13q14 deletion, are of increasing importance [6]. More recently, also genome sequencing, miRNA expression profiling and methylome studies are starting to offer new insights in the disease onset and progression. By the same token, specific epigenetic modifications together with protein alterations became valuable targets in leukemia research due to their reversible character and thus potential in therapy [7].

To extend the knowledge on CLL pathology and to identify new biomarker candidates, we applied quantitative mass spectrometry strategies to target the lower abundant proteins and peptides on patient and control samples [3]. The expressional differences between isolated age-matched healthy B-cells and CLL B-cells clearly showed that the morphological differences inherent to cancerous cells challenge disease marker discovery. Our comparative proteome analysis of UM and M CLL B-cells however, revealed that remarkably, only a limited amount of the identified proteins was differentially expressed between patients with a different outcome. For both the UM and M patient group, known up-regulations of proteins contributing to cell proliferation were corroborated [8,9].

Analysis of the iTRAQ (isobaric tag for relative and absolute quantitation) data at the peptide level surfaced an interesting aberrant proteolytic product of a histone protein: clipping of the histone H2A *C*-tail. The specific clipping of histone H2A after V_114_ (cH2A) was previously reported in leukemia and leukemia cell lines and is catalyzed by the so-called “H2A specific protease” (H2Asp) [10,11,12,13,14]. Recently, we identified the enzyme Neutrophil Elastase (NE) as an important candidate for the identity of the H2Asp [15]. Even though proteolysis is often not considered as a regulated PTM and the importance of protein degradation in biological functions is frequently unclear, new technologies have started to unravel the critical role of clipping in cellular homeostasis and disease [16,17]. In some reports, histone clipping has even been suggested to be a functional modification with epigenetic potential [18,19]. More specifically, cH2A caught our attention as H2A is the only histone with a *C*-terminus protruding out of the nucleosomal core and the clipping site is localized at the entry and exit points of the DNA [20]. In general, histone modifications help in determining the heritable transcriptional state and lineage commitment development in normal B-cells [21]. Consequently, we persisted in the investigation of the H2A clipping in CLL as disruption of the histone code is suggested to drive hematopoietic cells in lymphomagenesis [22]. Here, we initially observed that H2A clipping was more abundant in CLL patients compared to healthy controls while no differences were found between M and UM. However, we showed this was not due to the disease itself, since this clipping seemed to be associated with the amount of myeloid cells present in the predominantly lymphoid samples. To further unfold the actual role of histone H2A proteolysis, we examined this cH2A clipping during induced differentiation of myeloid THP-1 cells into macrophages through quantitative mass spectrometry [12]. We concluded that synchronization of the THP-1 cells before the stimulation abrogates the temporally uprise of H2A clipping which has initially been observed at the onset of the differentiation.

## 2. Results

### 2.1. Differential Protein Expression between Healthy B-Cells and Chronic Lymphocytic Leukemia (CLL) B-Cells of M^−^ and UM^+^ Staged Patients

The difference in protein expression between the B-cells of patients with indolent mutated ZAP70^−^ (M^−^) CLL, the aggressive unmutated ZAP70^+^ (UM^+^) CLL and healthy 50+ donors was quantified using iTRAQ. The six runs were merged into a dataset which comprised 18,014 MS/MS (mass spectrometry) queries, yielding 7473 annotated peptides which were derived from 536 unique proteins (Table S1a). For the ratios included in the analysis, in total 107 proteins differed significantly between groups (Table S1b). Among the leukemia samples, only 22 proteins were distinct between M^−^ and UM^+^ as opposed to 70 between healthy and both CLL samples. One label in each run comprised the same pool of all CLL samples, which was included to simplify inter-run comparison and to increase sensitivity.

Functional clustering annotation of the protein lists from differentially expressed proteins between healthy and leukemia B-cells, showed that most up-regulated proteins in the leukemia samples (both UM^+^ and M^−^) are involved in mRNA processing, implying an increased transcriptional activity in cancer cells (Table S1c). Compared to healthy, most of the proteins that are down-regulated in the leukemia cells are predominantly connected to actin binding and cellular localization [23]. A cellular component analysis confirmed that 64% of the proteins up-regulated in CLL are categorized as nuclear whereas down-regulated proteins are primarily located in the cytosol (60%) (Table S1c) [24]. These results suggest morphological differences between the healthy and cancerous cells rather than true molecular aberrancies.

In the limited list of proteins specifically overexpressed in the aggressive UM^+^ compared to M^−^ two proteins are involved in ATP-binding, one is a ribosomal subunit and the H2B and Prohibitin proteins are involved in chromosomal organization (Table 1). For proteins significantly down-regulated in UM^+^/M^−^, the Interleukin enhancer-binding factor 3 and Bcl-2-associated transcription factor 1 are associated with DNA but most proteins are involved in metabolic mechanisms or are cytoskeletal.

### 2.2. Quantitative Mass Spectrometry and Western Blot Analysis on CLL Samples Endorsed the Myeloid Origin of H2A Clipping

**Figure 1:**
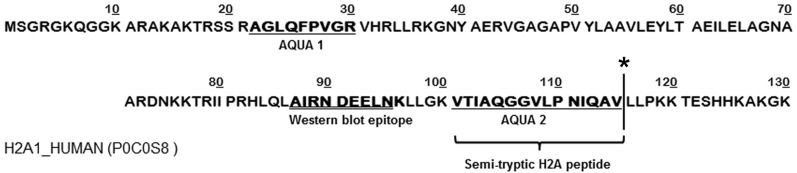
Sequence of histone H2A. Sequence from Uniprot [25]. The VTIAQGGVLPNIQAV peptide (*m*/*z* 740.4, charge 2+) was the only semi-tryptic peptide identified in all six runs. * indicates the clipping site after V_114_ (here described as amino acid 115 since methionine, generated by the start codon, is the first amino acid in the Uniprot sequence); Bold underlined sequences are the same as the isotopically labeled absolute quantification (AQUA) peptides, used for the subsequent specific cH2A quantitation. The bold double underlined sequence is the Western blot epitope.

Additional in-depth analysis at the peptide level, surfaced one remarkably aberrant modification in all the samples of the described iTRAQ analysis: clipping of histone H2A after V_114_ (Figure 1). The peptide VTIAQGGVLPNIQAV (*m*/*z* 740.4, charge 2+) was the only semi-tryptic peptide out of the >7400 annotated MS/MS spectra that was identified in all six runs. The annotation of the peptide was confirmed by *de novo* sequencing on the MS/MS spectrum (Figure S1). These results highlight how iTRAQ chemistry contributes to a better annotation of semi-tryptic peptides by enhancing the sensitivity due to the multiplexing and increased *b*-ion formation.

**Table 1:** Proteins which are differentially expressed between UM^+^ and M^−^ Chronic Lymphocytic Leukemia (CLL) B-cells. The table lists the protein name, Uniprot_ID, average UM^+^/M^−^ ratio, the number of iTRAQ samples where the protein was identified (*n*), the *p*-value of the performed *t*-test and the protein function (derived from UniProt GO).

UM^+^ *vs.* M^−^	Protein Name	Identified Protein	UM^+^/M^−^ Average Ratio	*n*	*p*-Value	Function (GO)
Down-regulated in UM^+^/M^−^	Serine/arginine-rich splicing factor	SRSF1_HUMAN	0.61	4	2.77 × 10^−4^	RNA binding
		SRSF3_HUMAN	0.77	5	1.30 × 10^−2^	RNA binding
		SRSF7_HUMAN	0.90	3	2.70 × 10^−2^	RNA binding
	Bcl-2-associated transcription factor	BCLF1_HUMAN	0.69	3	3.84 × 10^−2^	DNA binding, induction of apoptosis
	40S ribosomal protein	RS21_HUMAN	0.70	3	3.64 × 10^−2^	Ribosomal subunit
		RS28_HUMAN	0.67	4	1.47 × 10^−2^	Ribosomal subunit
	Stress-induced-phosphoprotein	STIP1_HUMAN	0.73	3	4.68 × 10^−2^	Golgi apparatus
	Protein S100-A6	S10A6_HUMAN	0.75	5	2.24 × 10^−2^	Calcium ion binding
	Heat shock cognate 71 kDa protein	HSP7C_HUMAN	0.79	5	1.44 × 10^−2^	ATP binding
	Matrin-3	MATR3_HUMAN	0.80	3	1.85 × 10^−2^	RNA binding
	Ezrin	EZRI_HUMAN	0.84	4	3.32 × 10^−2^	Actin filament
	Transitional endoplasmic reticulum ATPase	TERA_HUMAN	0.84	5	1.13 × 10^−2^	ATP binding
	Interleukin enhancer-binding factor	ILF3_HUMAN	0.87	3	5.81 × 10^−3^	DNA binding
	Heterogeneous nuclear ribonucleoprotein	HNRPK_HUMAN	0.88	6	1.38 × 10^−3^	ATP binding, RNA binding
	Heterogeneous nuclear ribonucleoprotein	ROA1_HUMAN	0.89	5	1.15 × 10^−2^	mRNA splicing
Up-regulated in UM^+^/M^−^	40S ribosomal protein	RS13_HUMAN	1.27	5	4.81 × 10^−2^	Ribosomal subunit
	Prohibitin	PHB_HUMAN	1.35	4	3.72 × 10^−2^	DNA replication
	Endoplasmin	ENPL_HUMAN	1.80	3	2.05 × 10^−2^	ATP binding
	ATP synthase subunit beta, mitochondrial	ATPB_HUMAN	1.93	3	3.72 × 10^−2^	ATP binding
	Histone H2B	H2B1C_HUMAN	2.59	3	1.24 × 10^−2^	DNA binding
		H2B1D_HUMAN	1.10	3	1.84 × 10^−2^	DNA binding
		H2B1O_HUMAN	1.18	3	2.97 × 10^−2^	DNA binding

**Figure 2:**
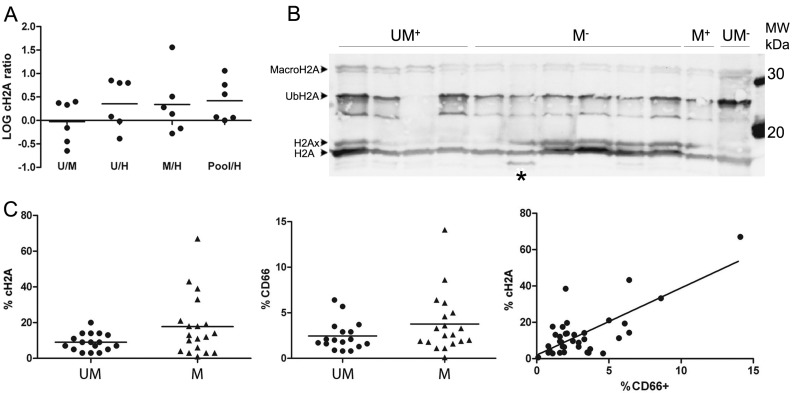
Quantitative mass spectrometry and Western blot analysis on CLL samples revealed that histone H2A clipping is from myeloid origin. (**A**) The iTRAQ ratios of the cH2A peptide (normalized to the average of the H2A protein to compensate for morphological differences) hint towards an increased abundance in CLL compared to healthy B-cells. The individual log ratios of all six runs are presented as dots, the average ratios as the horizontal bars. Histone H2A (cH2A) is up-regulated in the samples of the leukemia pool compared to the samples of the healthy B-cells in all six runs; (**B**) The histoneextracts from 12 of the 36 samples (From left to right: patient samples UM^+^ 8, 9, 10 and 11; M^−^ 9, 10, 11, 12, 13 and 14; M^+^ 5 and UM^−^ 6). Western blot with an H2A antibody against the epitope depicted in Figure 1 detects the H2A variants H2Ax, Ubiquitinated H2A and macroH2A. The band under ubiquitinated H2A could not be identified. cH2A was only faintly detected, except for one sample (*); (**C**) Specific cH2A screening with AQUA peptides and flow cytometry data revealed the myeloid characteristic of the clipping. **Left panel**: %cH2A differs significantly (*p* = 0.049) between CLL patients with a distinct mutational status (UM: 11× UM^+^ & 6× UM^−^, M: 14× M^−^ & 5× M^+^); **Middle panel**: Although not significant (*p* = 0.15), the %CD66b suggested a similar correlation with the mutational status; **Right panel**: Relation between %cH2a and %CD66b. Spearman’s Rho correlation between CD66b^+^ and %cH2A was significant at the 0.01 level (Spearman’s Rho correlation coefficient: 0.439; *p* = 0.007; Data: Table S2b).

For quantitation of this fragment, we compensated for abovementioned morphological differences by normalizing the reporter intensities of the clipping fragments to the average of the whole H2A protein. The relative amount of H2A that is cleaved after V_114_ was found to be on average higher in leukemia cells compared to healthy B-cells as seen by the log-transformed ratios. Particularly, all six ratios of the pool of UM^+^ and M^−^ samples were consistently positive opposed to healthy (Figure 2A).

**Figure 3:**
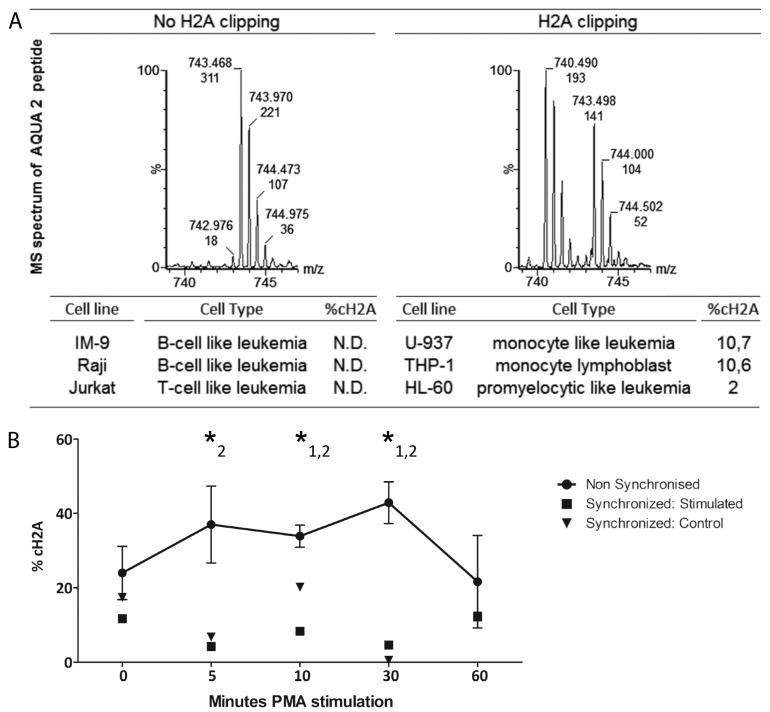
Specific cH2A quantitation in myeloid cell lines. (**A**) Example of an MS spectrum of the AQUA 2 peptide if H2A V_114_ clipping is absent (**left**) or present (**right**). The analysis of different cell lines confirms the myeloid characteristic of cH2A in the U-937, THP-1 and HL-60 cells. H2A clipping is not present in the investigated lymphatic cell lines and could not be unambiguously detected in the promyelocytic HL-60 cell line; (**B**) THP-1 cells stimulated with PMA show a transient H2A clipping pattern. Three biological replicates display the high variance. *1: significant differences of %cH2A between *T*_PMA0_ and the other data points; *2: the equivalent for *T*_PMA60_. The %cH2A of synchronized stimulated and control THP-1 cells, presented respectively as squares and triangles, is lower than the %cH2A of non-synchronized cells.

The consequences of proteolytic PTMs are ubiquitously underappreciated so we persisted in the more specific investigation of cH2A in a larger patient population due to the biomarker potential of this previously reported modification (Scheme of workflow: Figure S2). Immunodetection of H2A on histone extracts visualized cH2A only in one sample (Figure 2B, SYPRO staining: Figure S3). The flow cytometry data showed that this particular sample had the highest amount of granulocytes (expressed as %CD66b^+^ cells) in the screened patient population. In line with this, direct comparison of %cH2A with the more sensitive and specific AQUA (absolute quantification) approach suggested higher clipping in the M compared to UM CLL B-cells (Figure 2C, left panel) where a similar relationship between the amount of granulocytes cells and the respective mutational status was observed (Figure 3C, middle panel). Indeed, a statistical analysis of the possible correlation between the amounts of V_114_ clipped H2A and all cellular markers investigated for each sample confirmed a significant correlation between the amount of CD66b^+^ cells present in the samples and %cH2A (Figure 3C, right panel) (Details patient screening are listed in Table S2). Even though all steps were performed at 4 °C in the presence of protease inhibitors, we observed that H2A clipping can actually be caused *in vitro* to some extent as we observed some residual clipping activity on spiked-in biotinylated H2A (data not shown).

### 2.3. Characterization of cH2A in THP-1 Cells

To further corroborate the myeloid nature of cH2A we cultured human leukemia cell lines of different origin and prepared histone extracts when a density of approximately 1 × 10^6^/mL was achieved. In all the investigated lymphatic cell lines, no clipping of H2A was detected. For the myeloid cells, ±10% cH2A was measured in the monocyte-like THP-1 and U-937 cell lines and, although not unambiguously, ±2% in the promyelocytic HL-60 cells (Figure 3A).

Next, we applied our sensitive and specific AQUA approach to specifically quantify H2AV_114_ clipping in a previously reported model in which Ohkawa *et al.* describe a temporal increase of truncated H2A proteins during stimulated differentiation of acute monocytic leukemia THP-1 cells into macrophages using both phorbol 12-myristate 13-acetate (PMA) and retinoic acid (RA) [13]. For three biological replicates, %cH2A was specifically quantified in histone extracts, isolated at different time-points shortly after PMA supplementation (Figure 3B). The clipping at *T*_PMA0_ was significantly different than at *T*_PMA 10_ (*p* = 0.045) and *T*_PMA60_ (*p* = 0.011). Equally as to *T*_PMA60_, %cH2A was significantly different than at *T*_PMA 5_ (*p* = 0.041), *T*_PMA 10_ (*p* = 0.010) and *T*_PMA 30_ (*p* = 0.0075). These results confirm that H2A clipping at V_114_ indeed ascends and sequentially decreases upon THP-1 differentiation.

Finally, since we hypothesized that the variation might be due to differences in cell cycle synchronization, THP-1 cells were synchronized by double thymidine block, as the more open chromatin structure during cell division could very well be more susceptible to proteolytical activity during cell lysis. After synchronization, cells were either left untreated or were subjected to differentiation and cH2A was subsequently quantified by AQUA screening on histone extracts, obtained from different time points (Figure 3B). %cH2A was significantly lower in both the control (*p* = 6.09 × 10^−4^) and stimulated (*p* = 9.18 × 10^−5^) THP-1 cells compared to the unsynchronized cells. Further, no difference was observed between the synchronized stimulated and control cells (*p* = 0.22). Sampling 48 h after the start of the differentiation indicated that the amount of clipped H2A was further reduced in both cell lines (data not shown).

## 3. Discussion

Quantitative proteomics on patient samples and on leukemia cell lines can help to define new biomarkers and insights into the pathogenesis of lymphoid and hematopoietic neoplasms [2,3]. In our comparative proteome study between healthy and CLL B-cells of patients with different prognosis, we implemented two quantitative label-based mass spectrometry methods: iTRAQ and AQUA. The isobaric character of the iTRAQ labels allows multiplexing different samples, resulting in an increased signal and lower sample complexity [26]. We included both CLL samples of patients with a different disease prognosis and healthy B-cells in the analysis to obtain deeper insights in disease biology. A substantial part of the observed significant differences in relative protein expression between healthy and CLL B-cells is primarily due to differences in morphology between healthy and neoplastic cells [27]. Cancerous B-cells are indeed characterized by a larger nucleus and a denser cytoplasm, which we affirmed here by functional grouping and cellular component analysis of the up- and down-regulated proteins. We caution for the interpretation of proteomics data when healthy and malignant cells differ strongly in their morphology, an important restriction that is not always validated prior to relative comparison in proteomics approaches. From a clinical perspective however, aberrant ratios between patient groups with UM^+^, who have a bad prognosis and poor overall survival range and the less aggressive M^−^, are more relevant. Notably, less than 5% of the identified proteins were significantly different between these two groups. In this list of proteins with a log ratio significantly different from zero, defining distinctions between M^−^ and UM^+^, one of the most remarkable candidates is the Bcl-2-associated transcription factor 1 (*p* = 3.84 × 10^−2^). The Bcl-2 family regulates apoptosis and is an established hallmark in CLL as aberrant expression of Bcl-2 proteins causes apoptosis resistance of CLL B-lymphocytes. Although not all the Bcl-2 proteins correlate consistently with known CLL biomarkers, several Bcl-2-anatagonists are in clinical trials for CLL treatment [8].

The use of the iTRAQ label and the subsequent manual analysis of the results at the peptide level together surfaced another fascinating finding: a semi-tryptic peptide derived from histone H2A clipped at V_114_. Of all the trypsin-based mass spectrometry experiments uploaded in the PRIDE database, this fragment was only annotated once (accession: 10,528) [28]. Remarkably, we identified the peptide in all six runs. This could be explained by the contribution of the iTRAQ labels which are covalently bound to the peptide *N*-termini, generating intense b-series and consequently contributing to an in increased peptide score [26]. cH2A is generated by the removal of fifteen amino acids from the carboxy-terminal end of the intact H2A molecule after V_114_, coordinately removing K119 which is an important site of mono-ubiquitination [29,30,31,32,33]. cH2A had been described over 35 years ago as a product of H2A specific protease (H2Asp) activity [14] and shortly afterward, other groups observed similar cleavage patterns in extracts from both myeloid and lymphatic leukemia’s [10,11]. Since then, this clipping event was only referenced on a few occasions.

Although the biological function of protein degradation is largely unknown, proteolysis is an important category of PTMs, e.g., 5%–10% of all drug targets are proteases [16]. Truncation of histone tails has already been linked to cell differentiation and the *C*-tail of H2A is known to be important in cellular homeostasis and chromatin biology [18,20]. We recently identified the myeloid enzyme NE as being a prime candidate to fulfill the reaction mediated by the H2Asp but could not clarify if cH2A formation is involved in an epigenetic process or is rather a consequence of NET formation [15]. In healthy hematopoietic cells we only observed H2A clipping in cells of myeloid origin but the references to clipped H2A found in literature are mainly in the context of leukemia [10,11,12,13]. We thus persisted in a more detailed analysis of this modification and examined if cH2A formation is an epigenetic hallmark of CLL.

The preliminary iTRAQ results indicated a greater average abundance of the cH2A peptide in leukemia samples compared to the healthy controls. As validation is required, we specifically quantified cH2A in histone extracts derived from CLL B-cells of 36 clinically staged patients by applying the isotopic synthesized AQUA peptides that allow to target specific known H2A peptides, present in low concentrations [26]. We could this time not define any direct connection between %cH2A and any known disease marker. However, the amount of granulocytes in the samples did correlate with %cH2A, which corroborates the myeloid character of cH2A and is in line with the identification of NE. As complete inhibition of proteases is known to be very challenging, as also seen by us in a spike-in experiment of biotinylated H2A (not shown), it is difficult to define how much, if any, cH2A is endogenously generated [34].

On the other hand, the reported transient clipping of the H2A *C*-tail during the induced differentiation of THP-1 promonocytes, implies a potential biological role of H2A processing in the hematopoietic development of cells from myeloid origin [13]. Cells may for instance have mechanisms to control histone degradation for re-establishing the epigenetic marks on their tails in the proliferating state [35]. However, although our AQUA results demonstrate a brief uprise in specific H2A V_114_ clipping upon PMA or RA stimulation, synchronization of the THP-1 cells before the stimulation abrogated such trend. Histone clipping seems to correlate with the myeloid cell cycle and as our results suggest, cH2A fluctuation is more likely caused by possible differences in cell cycle stage synchronization, rather than PMA or RA induced differentiation. Instead of being a regulated mechanism, we hypothesize that the high degree of variation found in these experiments probably is due to the more open chromatin structure during cell division rendering histones more susceptible to proteolytical activity.

## 4. Experimental Section

### 4.1. Cells and Reagents

Phosphate buffered saline (PBS), media, l-glutamine, Fetal bovine serum (FBS), penicillin/streptomycin, Dynabeads and SYPRO Ruby were from Life Technologies (San Diego, CA, USA), ammonium bicarbonate (ABC), sodium dodecyl sulfate (SDS), *N*-cyclohexyl-3-aminopropanesulfonic acid (CAPS) and Tween-20 from Millipore (Billerica, MA, USA). ReadyPrep sequential extraction kit was from BioRad (Hercules, CA, USA) and Vivaspin-2 columns from Sartorius (Göttingen, Germany). The Recombinant human H2A (M2502S) was obtained from New England Biolabs (Ipswich, MA, USA) and bovine histone extract (cat. no. 223565) from Roche (Basel, Switzerland). All other reagents were purchased from Sigma Aldrich (St. Louis, MO, USA) unless described otherwise. Raji, Jurkat and HL-60 cells were cultured in Dulbecco’s Modified Eagle Medium and IM-9, U-937 and THP-1 cells in RPMI-1640 medium, both enriched with 2% (*w*/*v*) l-glutamine, 10% (*w*/*v*) FBS and 50 IU/mL penicillin/streptomycin. To achieve synchronization at the late G1− early S phase, 2 mM thymidine was added over two intervals of 12–16 h, with an incubation in non-thymidine containing RMPI-1640 medium for 8 h in between 50 ng/mL PMA was added to the THP-1 medium for the differentiation of the non-synchronized cells and 1 µM RA for the differentiation of the synchronized cells.

### 4.2. Patient Samples

For the iTRAQ analysis, whole blood of six UM^+^ and six M^−^ clinically staged CLL patients was obtained from Ghent University Hospital, Department of Hematology. Informed consent was given according to the requirements of the Ethics Committee of the Ghent University Hospital. PBMCs were isolated from a Ficoll-Paque (GE Healthcare, Waukesha, WI, USA) density gradient, B-cells were purified using Dynabeads Untouched Human B (Life Technologies, San Diego, CA, USA). Correspondingly, control samples were isolated from healthy volunteers aged 50+. For the high throughput screening of cH2A, samples were obtained from the UCSD CLL Research Consortium (CRC). Immediately after thawing, cells were washed twice with cold PBS containing 1 mM phenylmethanesulfonyl fluoride (PMSF) and protease inhibitor cocktail (Roche, Basel, Switzerland). CD5^+^CD19^+^ cells consistently made out more than 85% of the lymphocyte population as seen by flow cytometry.

### 4.3. Flow Cytometry

For each measurement 2 × 10^5^ cells were washed twice at 4 °C with PBS 1% Bovine Serum Albumin (BSA) and analyzed using a Cytomics FC500 flow cytometer (Becton Dickinson Immunocytometry Systems, San Jose, CA, USA) with monoclonal antibodies (mAbs) antibodies from BD-Biosciences (Franklin Lakes, NJ, USA): Isotype controls, anti-CD5 (PECy5), anti-CD19 (FITC), anti-CD33 (PECy5), anti-CD66b (FITC) and anti-Annexin V (FITC). The synchronization of THP-1 cells was monitored with Propidium Iodide staining.

### 4.4. Cell Lysis and Histone Isolation

All steps were performed at 4 °C. To obtain a complete cell lysate for the iTRAQ analysis, cells were washed twice with PBS, pelleted and resuspended in the ReadyPrep sequential extraction buffer 1 at 5 × 10^6^ cells/mL, supplemented with protease inhibitor cocktail (Roche), 1 mM PMSF, 10 µL 200 mM Tributylphosphine (Biorad), 20 µL phosphatase inhibitor cocktail 1 and 2 and 1 μL 250-units/µL benzonase. After sonication and centrifugation at 1500 rpm for 5 min, the proteins in the supernatant were transferred to a new eppendorf. The obtained pellet was resuspended in buffer 3 from the extraction kit and sonicated for 10 min. After centrifugation at 1500 rpm for 5 min the supernatant was pooled with the previous extract. Detergents, inhibitors and urea were removed by washing twice with Milli-Q water on a Vivaspin-2 column.

For the histone extracts, harvested cells were washed twice in PBS containing 1 mM PMSF, and protease inhibitor cocktail. 10^7^ cells/mL were resuspended in Triton extraction buffer (PBS containing 0.5% (*v*/*v*) Triton 100×, 1 mM PMSF and protease inhibitor cocktail) and lysed by gentle stirring. Pelleted nuclei were subsequently washed in PBS containing 1 mM PMSF and proteinase inhibitor cocktail. Histones were extracted overnight after benzonase treatment of the sonicated nuclei by acid extraction: incubation in 250 µL 0.2 M HCl at 4 °C with gentle stirring. Precipitated proteins were pelleted and the supernatant containing the histones was dried and stored at −20 °C until further use. The Bradford Coomassie Assay determined the protein content of all samples.

### 4.5. Western Blot Analysis

An amount of 3 µg of a dried histone extract was suspended in Laemmli buffer and run on a 15% PreCast Gel (Biorad) for 30 min at 150 V and 60 min at 200 V and subsequently transferred to a nitrocellulose membrane in a 10 mM CAPS buffer with 20% MeOH (Merck, New York, NY, USA). The remaining proteins in the gel were visualized after overnight Sypro Ruby staining. For Western blot, all steps were performed at room temperature with intermediate washing steps in 0.3% Tween-20 in PBS. The Histone H2A (LS-C24265, LifeSpan BioScience, Seattle, WA, USA) antibody incubation was performed overnight in a 1:1000 dilution in PBS 1% BSA, followed by 1 h incubation in the same buffer, with a stabilized horseradish-peroxidase (HRP)-conjugated goat anti-rabbit immunoglobulin G (Pierce, Rockford, IL, USA). The Supersignal West Dura Extended Duration Substrate (Pierce) was applied to perform the chemiluminescence and both the gel and Western blot membrane were visualized with a VersaDoc Imaging System.

### 4.6. Quantitative Mass Spectrometry Analysis

All digests were performed according to the iTRAQ (ABSciex, Foster City, CA, USA) reagent Kit guidelines, as was the given labeling itself. For the iTRAQ analysis, six 4plex runs each encompassed 4 × 100 µg of the protein lysates from CLL B-cells from UM^+^, M^−^ and B-cells from healthy donors aged 50^+^. The fourth label was used for an additional pool of all 12 CLL samples for inter-run comparison and to increase the number of identifications. Technical variation was also minimized by reversing the labeling order. All six samples were first fractionated off line into 12 fractions on a Poros 10S strong-cation exchange (SCX) column (300 μm i.d. × 15 cm, ABSciex, Foster City, CA, USA) for subsequent nano reversed phase liquid chromatography (Dionex U3000, Dionex, Chelmsford, MA, USA) separation on a gradient specifically optimized for sample content of each fraction [36]. All other samples were separated on a 70 min organic gradient from 4%–100% buffer B (80% (*v*/*v*) acetonitrile (Millipore, Billerica, MA, USA) in 0.1% (*v*/*v*) FA). All samples were analyzed on an Electrospray ionization—Q-TOF Premier (Waters, Wilmslow, UK). Data was searched against the SwissProt (541,954 sequence entries) database using Mascot 2.3 and additionally manually interpreted with Mascot Distiller (Matrix Science, London, UK). Proteins were included for the analysis if a proteotypic peptide had a Mascot score above 45 in at least 3 runs. For iTRAQ quantitation, ratios were normalized based on summed intensities and only proteins recurring in at least three out of six different runs were withheld by an automated in-house approach. Gene ontology (GO) analysis was performed on the significantly up- or down-regulated proteins with Uniprot, DAVID and the WEB-based GEne SeT AnaLysis Toolkit (WEBGESTAT) [23,24,25].

For specific cH2A screening, a mix of the isobaric peptides (AQUA1, Thermo, Waltham, MA, USA) AQUA2, Sigma Aldrich (St. Louis, MO, USA) was added right before MS analysis. The tryptic H2A *N*-terminal peptide R.AGLQFPVGR.V (*m*/*z* 475.7 was used to quantify the total amount of histone H2A present in the sample (AQUA 1). Specific clipping is quantified by means of the semi-tryptic peptide K.VTIAQGGVLPNIQAV.L (*m*/*z* 743.4) (AQUA2). This is the same sequence as was found during the iTRAQ analysis. To 1 µg of HE, 10 pmol of AQUA1 and 1 pmol of AQUA 2 was spiked right before the MS analysis [15]. For the quantitation, the total ion current (TIC) from both AQUA peptides was obtained from a single MS-scan acquired on the top of the two extracted-ion chromatograms. An example of the MS spectra of the two AQUA peptides is given at the right site of the accolade in Figure S2. To confirm the efficiency of the histone extraction, the raw data of the data-directed analysis was equally searched against the SwissProt database using Mascot 2.3 (Matrix Sciences, London, UK).

### 4.7. Statistical Analysis

For the iTRAQ analysis, ratios were log transformed and averaged. Statistical analysis was performed by means of a homoscedastic two-tailed *t*-test to inspect which log ratios were significantly different from zero (*p* = 0.05). The average ratio of a protein indicated whether a protein is up- or down-regulated between two samples. The same *t*-tests were performed to test if %cH2A and %CD66b was different between the M and UM patients. For the comprehensive analysis of the AQUA screening, the relationship between cH2A and the other variables was determined by the nonparametric Spearman’s Rho correlation test wherein the ZAP and the mutational status were analyzed as dichotomized values (+ or −) with the SPSS Statistics 20 software (Endicott, NY, USA). To corroborate if the clipping temporally increased during the differentiation of the THP-1 cells, one-tailed *t*-tests were applied to compare each data point with the %cH2A at T_PMA 0_ and T_PMA 60_. With the same test, all the data points of the non-synchronized experiment were examined against all the data points of the synchronized (stimulated or control) experiment. Similarly, a *t*-test was used to validate if the %cH2A between the synchronized stimulated and control cells was analogous.

## 5. Conclusions

We particularly emphasize both the pitfalls and the benefits of applying quantitative proteomic strategies in hematological research. Although morphological differences hamper proteome-wide comparison of healthy and leukemia B-cells, we resurfaced a previously described clipping product of the H2A *C*-tail. A more profound characterization in CLL and THP-1 samples indicates that H2A V_114_ clipping occurs in hematopoietic cells of the myeloid lineage. Although the process of histone clipping has considerable epigenetic potential, many questions about the relevance of specific histone proteolysis remain.

## Supplementary Files

Supplementary File 1 Supplementary Materials (ZIP, 1965 KB)
