# Supplementary material for: Quantitative Proteomics to Characterize Specific Histone H2A Proteolysis in Chronic Lymphocytic Leukemia and the Myeloid THP-1 Cell Line
Source: Int J Mol Sci. 2014 May 27;15(6):9407–21. doi: 10.3390/ijms15069407 (PMC4100102; doi:10.3390/ijms15069407)
Supplement: Supplementary File 1 — Supplementary Materials (ZIP, 1965 KB) [file ijms-15-09407-s001.zip › ijms-50646-Figures S1-S3.pdf]

Supplementary Information

Figure S1. Validation of the annotation of the semi-tryptic peptide VTIAQGGVLPNIQAV on the raw data of the MSMS spectrum in Mascot Distiller (Matrix Science). **Upper panel**: The iTRAQ-labeled H2A peptide. The Mascot score of the labeled peptide is 70 and the expectation value is 2×10^{-6}; **Lower panel**: *De novo* sequencing of the non-labeled H2A peptide. The Mascot score is only 40 with a expectancy value of 3×10^{-2}.

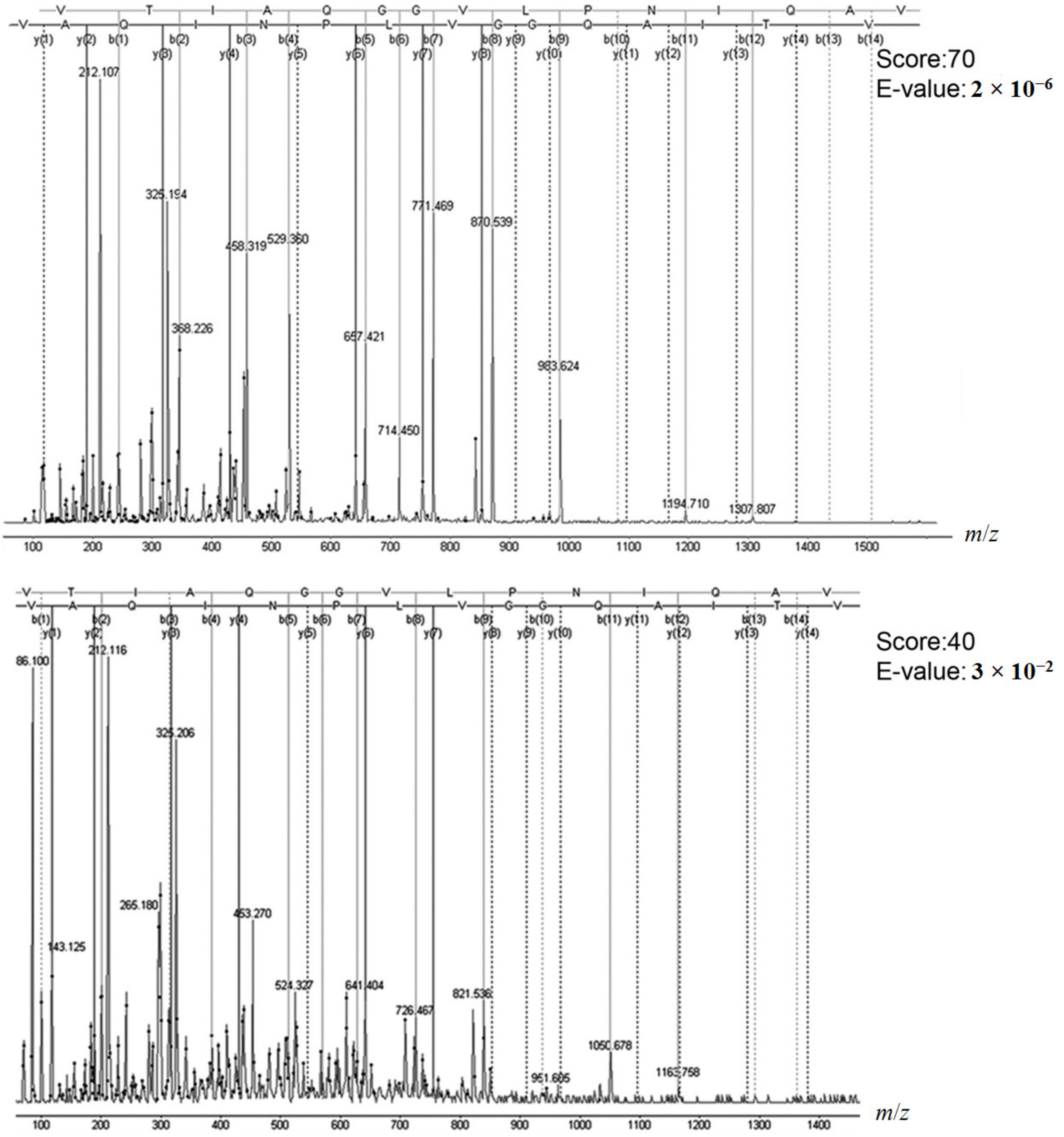

This difference could be explained by the contribution of the iTRAQ labels which are covalently bound to the peptide *N*-termini, generating more intense b-series and consequently resulting in an increased peptide score. For tryptic peptides, this effect is of minor importance, but when the peptide does not have the conventional K or R at y1, this significantly changes the fragmentation pattern and facilitates the automated identification.

Figure S2. Scheme of the workflow of the CLL screening on 36 different patient cells with a variable prognosis. Frozen cells are thawed and washed with PBS. Flow cytometry was performed for CD5:CD19, CD33:CD66b and AnnexinV: PI analysis on 5×10^5 cells. The rest of the cells were lysed and histones were extracted with acid. After protein quantitation, 3 µg histones was applied for gel electrophoresis and subsequent Western blot. 2 µg was digested and the AQUA peptides were added before LC-MSMS. **Right panel**: At the top of the elution peak as seen on the extracted ion chromatogram of *m*/*z* 474.8 2+, the amount of total H2A in the sample was quantified with the AQUA 1 peptide. The AQUA 2 peptide (*m*/*z* 743.4 2+) was spiked to quantify cH2A in order to subsequently calculate clipped H2A. $\%cH2A = ((740.4/743.4)_{TIC})/(10(470.7/473.7)_{TIC}) \times 100$.

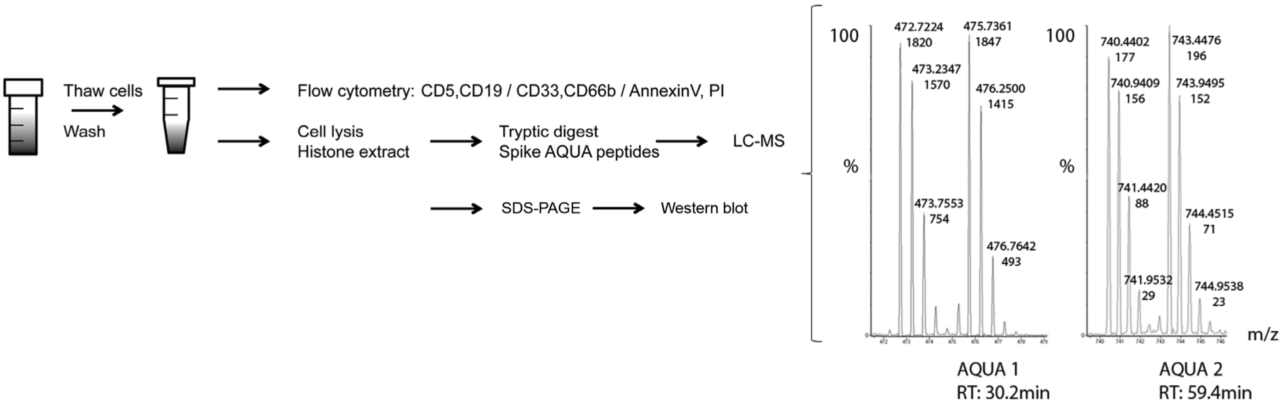

Figure S3. The histone extracts from 12 samples (From left to right: patient samples UM$^+$ 8, 9, 10 and 11, M$^-$ 9, 10, 11, 12, 13 and 14, M$^+$ 5 and UM$^-$ 6). Sypro staining of all the proteins demonstrate the purity of the HE. Clean bands of all the core histones were visualized together with the molecular weight marker in the exterior lane.

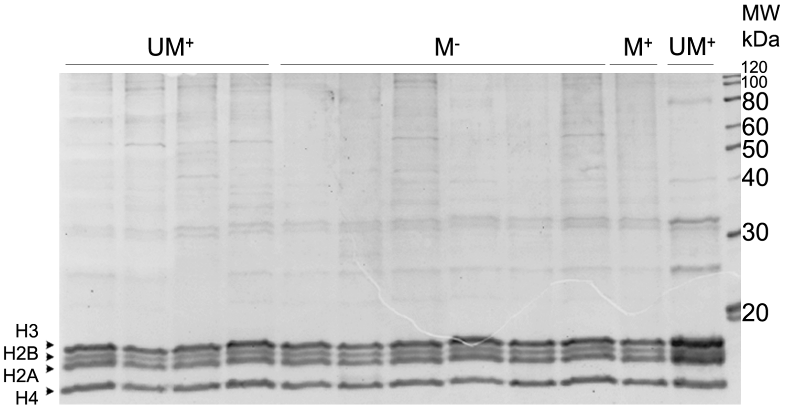

© 2014 by the authors; licensee MDPI, Basel, Switzerland. This article is an open access article distributed under the terms and conditions of the Creative Commons Attribution license (http://creativecommons.org/licenses/by/3.0/).
